# Supplementary material for: Evidence of Mitochondrial Dysfunction in Fibromyalgia: Deviating Muscle Energy Metabolism Detected Using Microdialysis and Magnetic Resonance
Source: J Clin Med. 2020 Oct 31;9(11):3527. doi: 10.3390/jcm9113527 (PMC7693920; doi:10.3390/jcm9113527)
Supplement: Supplementary file 1 [file jcm-09-03527-s001.pdf]

Supplementary Materials

MRS processing

The older software version of the MR-scanner was present when 42 subjects were examined whereof 21 each of the FMS and CON groups were examined; of these 6 each of the FMS and CON subjects were examined with the older MRS-coil. There was no significant difference (χ^2-test, p = 1.00) with respect to the use of the older coil between the groups. The newer software version was present when 20 subjects were examined, of which 11 were FMS subjects and 9 were CON subjects. There was no significant difference (χ^2-test, p = 0.71) with respect to the use of the newer software version of the MR-scanner between the groups.

Supplementary Table 1. OPLS of the three grip strength variables (i.e., three simultaneous Y-variables) in all subjects taken together. As regressors were used data from spectroscopy and microdialysis (except pain variables from microdialysis) (cf. Tables 3-4). The regression had one predictive component. Only significant variables are shown (i.e., variables with VIP > 1.0 and absolute p(corr) ≥0.50).

**Variables**	**VIP**	**p(corr)**
Mean Pyruvate Trapezius 140–220 min	1.79	−0.68
Blood flow Erector spinae 140 min	1.73	0.65
Blood flow Erector spinae 160 min	1.67	0.64
Mean Blood Flow Erector Spinae 140–220 min	1.66	0.63
Pyruvate Trapezius 140 min	1.63	−0.62
Mean Pyruvate Erector spinae 140–220 min	1.62	−0.61
ATP	1.51	0.58
Pi/PCr	1.49	−0.57
PCr	1.45	0.55
Mean Glycerol Erector spinae 140–220 min	1.42	−0.53
Pyruvate Erector spinae 160 min	1.41	−0.53
Mean Glycerol Trapezius 140–220 min	1.40	−0.53
Mean Blood flow Trapezius 140–220 min	1.35	0.51
PCr/P_{tot}	1.32	0.51
R^2	0.28	
Q^2	0.19	
CV-ANOVA *P* -value	0.002–0.013	
n	60	

VIP and p(corr) are reported for each regressor (i.e., the loading of each variable scaled as a correlation coefficient and therefore standardising the range from −1 to +1. The sign of p(corr) indicates the direction of the correlation with the dependent variable (+ = positive correlation; - = negative correlation). Hence, a positive p(corr) for a certain variable indicates a positive correlation with grip strength variables in the multivariate context. The four bottom rows of each regression report R^2, Q^2, *P*-value of the CV-ANOVA, and number of subjects included in the regression (n) from the second OPLS regression (see Statistics for details).

Supplementary Table 2. OPLS of TST in all subjects taken together. As regressors were used data are from spectroscopy and microdialysis (except pain variables from microdialysis) (cf. Tables 3–4). The regression had one predictive component. Only significant variables are shown (i.e., variables with VIP > 1.0 and absolute p(corr) ≥0.50).

Variables	VIP	p(corr)
Pi/PCr	1.94	−0.70
Mean Pyruvate Erector spinae 140–220 min	1.89	−0.68
Mean Pyruvate Trapezius 140–220 min	1.83	−0.65
Blood flow Erector spinae 140 min	1.77	0.64
Pyruvate trapezius 140 min	1.70	−0.60
Pi/P_{tot}	1.70	−0.61
Blood flow Erector spinae 160 min	1.64	0.60
Mean Blood Flow Erector Spinae 140–220 min	1.63	0.59
Pyruvate Erector spinae 160 min	1.61	−0.59
PCr/P_{tot}	1.60	0.58
Difference Pyruvate Erector spinae 160 min–140 min	1.48	−0.54
R^2	0.33	
Q^2	0.18	
CV-ANOVA *P*-value	0.005	
n	59	

VIP and p(corr) are reported for each regressor (i.e., the loading of each variable scaled as a correlation coefficient and therefore standardising the range from −1 to +1). The sign of p(corr) indicates the direction of the correlation with the dependent variable (+ = positive correlation; - = negative correlation). Hence, a positive p(corr) for a certain variable indicates a positive correlation with TST in the multivariate context. The four bottom rows of each regression report R^2, Q^2, *P*-value of the CV-ANOVA, and number of subjects included in the regression (n) from the second OPLS regression (see Statistics for details).

Supplementary Table 3. OPLS of TST in FM. As regressors were used data are from spectroscopy and microdialysis (except pain variables from microdialysis) (cf. Tables 3–4). The regression had one predictive component. Only significant variables are shown (i.e., variables with VIP > 1.0 and absolute p(corr) ≥0.50).

**Variables**	**VIP**	**p(corr)**
Mean Pyruvate Erector spinae 140–220 min	2.38	−0.91
Pyruvate Erector spinae 160 min	2.20	−0.85
Glutamate Erector spinae 160 min	2.14	−0.82
Glucose Erector spinae 160 min	2.10	−0.81
Difference Pyruvate Erector spinae 160 min–140 min	2.10	−0.81
Mean Pyruvate Trapezius 140–220 min	2.00	−0.76
Difference Glucose Erector spinae 160 min–140 min	1.93	−0.74
Mean Glutamate Erectors spinae 140–220 min	1.88	−0.72
Mean Glucose Erectors spinae 140–220 min	1.84	−0.71
Difference Glutamate Erector spinae 160 min–140 min	1.69	−0.65
Pi/P_{tot}	1.45	−0.56
Pyruvate trapezius 140 min	1.38	−0.52
R^2	0.29	
Q^2	0.21	
CV-ANOVA *P*-value	0.040	
n	31	

VIP and p(corr) are reported for each regressor (i.e., the loading of each variable scaled as a correlation coefficient and therefore standardising the range from −1 to +1). The sign of p(corr) indicates the direction of the correlation with the dependent variable (+ = positive correlation; - = negative correlation). Hence, a positive p(corr) for a certain variable indicates a positive correlation with TST in FM in the multivariate context. The four bottom rows of each regression report R^2, Q^2, *P*-value of the CV-ANOVA, and number of subjects included in the regression (n) from the second OPLS regression (see Statistics for details).

Supplementary Table 4. OPLS of the aerobic fitness in FM. As regressors were used data are from spectroscopy and microdialysis (except pain variables from microdialysis) (cf. Tables 3–4). The regression had one predictive component. Only significant variables are shown (i.e., variables with VIP > 1.0 and absolute p(corr) ≥0.50).

Variables	VIP	p(corr)
Difference Glycerol Erector spinae 160 min–140 min	1.85	0.62
Pyruvate Trapezius 160 min	1.79	−0.75
Lactate Trapezius 160 min	1.67	−0.70
Mean Pyruvate Trapezius 140–220 min	1.60	−0.67
Mean Glycerol Trapezius 140–220 min	1.58	−0.66
Lactate Erector spinae 160 min	1.54	−0.64
Glycerol Trapezius 160 min	1.51	−0.63
Mean Lactate Trapezius 140–220 min	1.38	−0.57
Glycerol Erector spinae 140 min	1.37	−0.57
Pyruvate trapezius 140 min	1.36	−0.57
Difference Pyruvate Trapezius 160 min–140 min	1.35	−0.56
Mean Glutamate Erector spinae 140–220 min	1.27	−0.53
Glutamate Trapezius 160 min	1.25	−0.52
Difference Glycerol Trapezius 160 min–140 min	1.24	−0.52
Mean Pyruvate Erector spinae 140–220 min	1.22	−0.51
Mean Glutamate Trapezius 140–220 min	1.21	−0.51
Glycerol Trapezius 140 min	1.21	-0.50
R^2	0.30	
Q^2	0.23	
CV-ANOVA *P*-value	0.033	
n	30	

VIP and p(corr) are reported for each regressor (i.e., the loading of each variable scaled as a correlation coefficient and therefore standardising the range from −1 to +1). The sign of p(corr) indicates the direction of the correlation with the dependent variable (+ = positive correlation; - = negative correlation). Hence, a positive p(corr) for a certain variable indicates a positive correlation with aerobic fitness for FM in the multivariate context. The four bottom rows of each regression report R^2, Q^2, *P*-value of the CV-ANOVA, and number of subjects included in the regression (n) from the second OPLS regression (see Statistics for details).

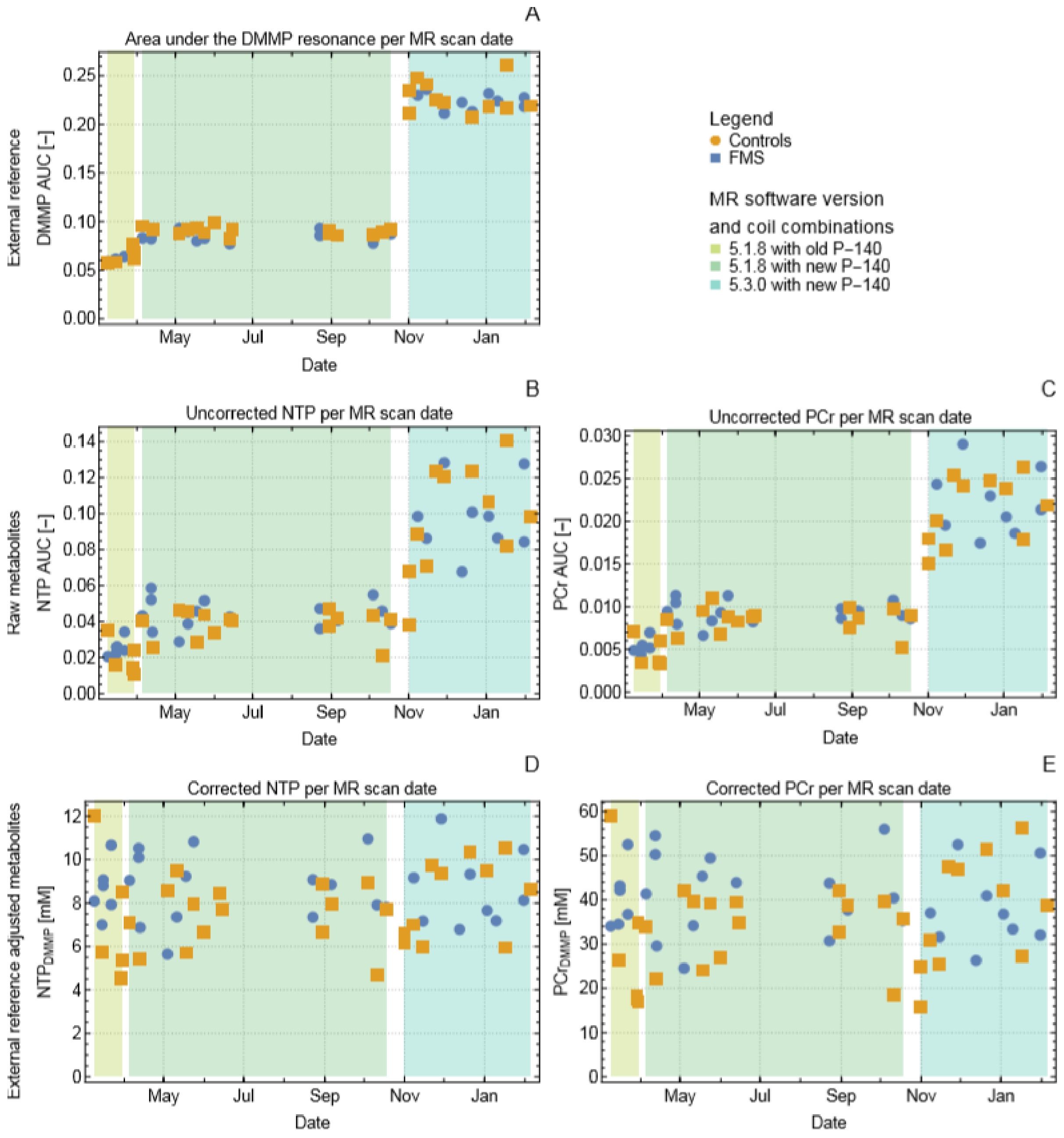

Figure 1. MRS Correction.
